# Engineering and Characterization of 3-Aminotyrosine-Derived Red Fluorescent Variants of Circularly Permutated Green Fluorescent Protein

**DOI:** 10.3390/bios14010054

**Published:** 2024-01-20

**Authors:** Hao Zhang, Xiaodong Tian, Jing Zhang, Hui-wang Ai

**Affiliations:** 1 Center for Membrane and Cell Physiology, University of Virginia, Charlottesville, VA 22908, USA; hz5qd@virginia.edu (H.Z.); xt3eg@virginia.edu (X.T.); jz4m@virginia.edu (J.Z.); 2 Department of Chemistry, University of Virginia, Charlottesville, VA 22904, USA; 3 Department of Molecular Physiology and Biological Physics, University of Virginia, Charlottesville, VA 22908, USA; 4 The UVA Comprehensive Cancer Center, University of Virginia, Charlottesville, VA 22908, USA

**Keywords:** nonconical amino acid, protein engineering, redox sensitivity, red-shifted fluorescence

## Abstract

Introducing 3-aminotyrosine (aY), a noncanonical amino acid (ncAA), into green fluorescent protein (GFP)-like chromophores shows promise for achieving red-shifted fluorescence. However, inconsistent results, including undesired green fluorescent species, hinder the effectiveness of this approach. In this study, we optimized expression conditions for an aY-derived cpGFP (aY-cpGFP). Key factors like rich culture media and oxygen restriction pre- and post-induction enabled high-yield, high-purity production of the red-shifted protein. We also engineered two variants of aY-cpGFP with enhanced brightness by mutating a few amino acid residues surrounding the chromophore. We further investigated the sensitivity of the aY-derived protein to metal ions, reactive oxygen species (ROS), and reactive nitrogen species (RNS). Incorporating aY into cpGFP had minimal impact on metal ion reactivity but increased the response to RNS. Expanding on these findings, we examined aY-cpGFP expression in mammalian cells and found that reductants in the culture media significantly increased the red-emitting product. Our study indicates that optimizing expression conditions to promote a reduced cellular state proved effective in producing the desired red-emitting product in both *E. coli* and mammalian cells, while targeted mutagenesis-based protein engineering can further enhance brightness and increase method robustness.

## 1. Introduction

Fluorescent proteins (FPs) have become indispensable tools for molecular imaging in live cells, offering a wide range of properties for various imaging applications [1,2]. Among these FPs, red fluorescent proteins (RFPs) have garnered significant attention due to their red-shifted excitation and emission spectra [3,4,5]. The red-shifted wavelengths of RFPs provide several advantages, including reduced phototoxicity, minimized autofluorescence, and increased tissue penetration for imaging deeper into biological samples [6,7,8].

Naturally occurring RFPs, such as DsRed [9] and anm2CP [10], have limitations in their application as imaging probes because they tend to be oligomeric and exhibit poor folding at physiological temperatures (37 °C). To overcome these challenges, extensive engineering efforts have been focused on monomerizing RFPs while simultaneously enhancing their brightness and chromophore maturation [11]. As a result, new RFP variants, including stagRFP [5] and mScarlet3 [12], have emerged with photophysical properties comparable to commonly used green FPs (GFPs) and yellow FPs (YFPs).

FPs have proven to be valuable tools for developing fluorescent biosensors, which play a crucial role in detecting and quantifying analytes or bioactivities [13]. Circular permutation is a commonly employed engineering strategy in biosensor development [14,15,16,17]. This approach involves modifying an FP by connecting its original N- and C-termini and creating new N- and C-termini near the chromophore responsible for fluorescence, resulting in circularly permuted FPs (cpFPs) [14,15,18]. Certain cpFP variants exhibit enhanced sensitivity to their surrounding environment, making them ideal signal output modules for biosensor applications. To further create a functional biosensor, a cpFP is fused with a sensory domain, which undergoes a conformational change in response to analytes or bioactivities. By coupling the sensing domain with cpFP, the conformational change triggered by analyte or bioactivity detection leads to a corresponding change in fluorescence signal [19].

While significant efforts have been dedicated to developing enhanced RFPs and engineering them into red fluorescent biosensors, alternative approaches have explored directly red-shifting GFPs and GFP-derived biosensors. Light-induced green-to-red conversion has been observed in photoconvertible fluorescent proteins [20,21,22,23], and partial red-shift of GFP emission has been achieved under specific conditions such as oxygen depletion, strong irradiation, or in the presence of an electron acceptor [24,25,26,27,28]. However, direct conversion of a typical GFP to an RFP proved challenging until a recent study, which successfully engineered GFPs into RFPs through extensive mutagenesis [29].

In parallel, the site-specific incorporation of noncanonical amino acids (ncAAs) has been employed to achieve red-shifts in GFPs and GFP-derived biosensors. This approach involves the use of orthogonal transfer RNAs (tRNAs) and aminoacyl-tRNA synthetases (aaRSs) to replace the chromophore-forming tyrosine of GFP with a designed ncAA [30,31,32,33]. Through this method, several ncAA-derived variants of GFP with red-shifted fluorescence have been successfully developed [34,35]. In previous studies, our research group and others have demonstrated the use of 3-aminotyrosine (aY), a specific ncAA, to modify the GFP chromophore [36,37], leading to consistent red-shifting of the spectra in GFPs, circularly permuted GFPs (cpGFPs), and GFP-based biosensors.

Despite the progress, we have encountered challenges that have resulted in inconsistent results. We postulated several factors contributing to these inconsistencies, including the redox and metal ion sensitivity of aY and aY-derived chromophore, and issues such as the existence of undesired green fluorescent species and the overestimation of the brightness of the red-emitting species [38]. These challenges have highlighted the need for further refinement and optimization of the method to ensure more reliable and consistent outcomes.

We have recently conducted a method optimization using aY-modified superfolder GFP (aY-sfGFP) [38,39]. Through our experiments, we discovered that expressing the protein in sealed culture containers resulted in purer protein samples with minimal green species [38]. Additionally, our collaborators employed femtosecond stimulated Raman spectroscopy and other techniques to investigate the properties of aY-sfGFP. The findings revealed that aY-sfGFP possesses a GFP-like chromophore, and the red color arises from a unique “double-donor” chromophore structure that elevates the ground-state energy and enhances charge transfer [38]. Furthermore, we developed mutants of aY-sfGFP, and a single E222H mutation improved the brightness 12-fold [38].

In this study, our main focus is on an aY-derived cpGFP (aY-cpGFP). We began by optimizing the expression conditions to improve protein production. Subsequently, we conducted mutagenesis studies to generate two variants of aY-cpGFP with enhanced brightness. Additionally, we investigated the responsiveness of these mutants to common metal ions, as well as reactive oxygen and nitrogen species (ROS and RNS). Through our comprehensive characterization, we aim to provide valuable insights into the performance and potential applications of aY-cpGFP mutants and fluorescent biosensors.

## 2. Materials and Methods

### 2.1. Materials, Reagents, and General Methods

All oligos (Supplementary Materials, Table S1) were purchased from Integrated DNA Technologies (San Diego, CA, USA). pEvol-MjaYRS (Addgene #153557 (Watertown, MA, USA)), pMAH-EcaYRS (Addgene #153558), and pBAD-pnGFP-Ultra (Addgene #157923) were previously reported by our laboratory [36,40]. The aY amino acid (Catalog #4027898) was purchased from Bachem (Torrance, CA, USA). Cell culture media and sera were purchased from Corning (Corning, NY, USA). All other chemicals and enzymes were purchased from Fisher Scientific (Hampton, NH, USA). DNA purification columns were purchased from Syd Laboratories (Malden, MA, USA). All modified DNA sequences were confirmed using Eurofins Genomics’s service (Louisville, KY, USA). ChatGPT (OpenAI, San Francisco, CA, USA) was used to rephrase certain sentences in this manuscript while preserving the original meaning of the content.

### 2.2. Plasmid Construction and Screening

Random mutagenesis was conducted using error-prone polymerase chain reactions (EP-PCRs) modified from a previously reported method [41]. Random errors were introduced in the presence of Taq DNA polymerase, 5 mM MnCl_2_, 25 mM MgCl_2_, and an unbalanced dNTP condition containing 1 mM of dCTP and dTTP, and 0.2 mM of dGTP and dATP. Site-directed mutations, including site-directed saturation mutagenesis, were performed using polymerase chain reactions with mutations introduced to the PCR primers (Table S1). The DNA fragments were purified using gel electrophoresis and column-based gel extraction, before being introduced into the pBAD-His B vector between the Xho I and Hind III restriction sites. For screening purposes, the gene library in the pBAD-His B vector was then used to transform DH10B electrocompetent *E. coli* cells containing pEvol-MjaYRS. The transformed *E. coli* cells were plated on 2YT agar plate supplemented with 100 μg/mL ampicillin, 100 μg/mL chloramphenicol, 2 mM aY, and 0.2% (*w*/*v*) L-arabinose. After incubation at 37 °C overnight, the plates were sealed with parafilm and incubated at room temperature for 48–72 h. The fluorescence of bacterial colonies was examined, and colonies with high red fluorescence were identified using an imaging setup (excitation filter 550/30 nm, emission filter 645/75 nm) described previously [42]. Selected clones were cultured individually in a sealed, oxygen-limited deep-96 well culture plate in 200 μL of Terrific Broth (TB) medium supplemented with 100 μg/mL ampicillin and 100 μg/mL chloramphenicol, shaking at 250 r.p.m. and 37 °C overnight (~18 h). Protein expression was induced by adding another 200 μL of 2xYT medium supplemented with 100 μg/mL ampicillin, 100 μg/mL chloramphenicol, 4 mM aY, and 0.4% (*w*/*v*) L-arabinose. The plate was sealed again to limit oxygen and cultured at 30 °C with shaking for additional 48 h. Cells were pelleted and then lysed using a laboratory-made solution containing 0.5% octyl glucoside, 0.1 mg mL^−1^ chicken egg lysozyme, and 0.2 U mL^−1^ Benzonase in 20 mM Tris-HCl, pH 8 on ice with gentle shaking for 30 min. Cell debris was pelleted, and the supernatants were used for testing on a BioTek Synergy Mx Microplate Reader. The most promising clones (high red fluorescence) were selected for further confirmation and characterization.

### 2.3. Cell Culture and Imaging

Human Embryonic Kidney (HEK) 293 T cells were cultured in 4.5 g/mL Dulbecco’s Modified Eagle’s Medium (DMEM) supplemented with 10% fetal bovine serum (FBS). Cells were incubated at 37 °C with 5% CO_2_ in humidified air. Cells seeded in 35 mm dishes were transfected at 40% confluency with 3 μg of total plasmid DNA (divided further equally when multiple plasmids were used) and 9 μg of PEI (Polyethylenimine, linear, M.W. 25 kD) in complete medium. After 24 h, the medium was replaced with DMEM containing 10% FBS and 2 mM aY. Fluorescence imaging was typically performed between 48 and 72 h post transfection. Before imaging, cells were rinsed once with room temperature DPBS and then equilibrated with mammalian cell imaging buffer (114 mM NaCl, 2.2 mM KCl, 22 mM NaHCO_3_, 1.1 mM NaH_2_PO_4_, 2 mM D-glucose, 2 mM CaCl_2_, 2 mM MgCl_2_, 25 mM HEPES, pH 7.4) for ~ 30 min. Images were acquired and analyzed as previously described [42]. Cells were manually circled, and mean fluorescence intensity within each region of interest was used for intensity analysis.

### 2.4. Protein Expression and Characterization

DH10B electrocompetent cells containing the pEvol-MjaYRS plasmid were transformed with the corresponding pBAD plasmids. A single colony was used to inoculate 3.0 mL TB supplemented with 100 μg/mL ampicillin and 35 μg/mL chloramphenicol in a cap-sealed 15 mL culture tube at 37 °C and 250 rpm overnight. Then, 1 mL of the saturated starter culture was added to 100 mL fresh TB supplemented with 100 μg/mL ampicillin and 35 μg/mL chloramphenicol in a 500 mL flask. The container was sealed and shaken at 37 °C and 250 r.p.m. for about 4 h. Once the bacterial growth reached the log phase, indicated by an OD_600_ (optical density at 600 nm) of 0.6, 0.2% L-arabinose and 4 mM aY were added to induce protein expression. The flask was sealed again and incubated at 30 °C and 250 r.p.m. for 48 h. Cells were pelleted, resuspended in 1 × PBS (phosphate-buffered saline, pH 7.4), and lysed by sonication. Ni-NTA agarose beads (Pierce, Rockford, IL, USA) were used to enrich and purify the His-tagged proteins. The eluted protein was further purified and characterized with a HiLoad 16/600 Superdex 200 pg size-exclusion column using an Akta protein purification system (Cytiva, Marlborough, MA, USA), and eluted in a buffer containing 150 mM NaCl and 30 mM Tris HCl, pH 7.4.

Absorption, fluorescence spectra, quantum yields, molar absorption, and pH sensitivity were characterized using a published protocol [36]. Fluorescent lifetime was determined using a Lambert Instrument (Groningen, Netherlands) LIFA-Toggel fluorescence lifetime imaging system. Specifically, 10 μL of each protein (500 nM) was placed in a 35 mm dish. To ensure proper focus, we used the droplet edge as a reference point. For excitation, we employed a 527 nm LED light source, a customized filter cube from Chroma Technology with a 530/30 nm bandpass excitation filter and a 574/40 nm bandpass emission filter, and an exposure time of 50 ms. The frequency was set to 40 MHz, and the phase number to 12. The fluorescence lifetime was determined using the LIFA software (version 1.3.0) with mScarlet-I (τ = 3.1 ns) used as the lifetime reference, and the fluorescence lifetime values reported in this work were derived from phase shifts. The redox reactivity analysis was conducted following the previously established protocol [40]. Briefly, a concentration of 500 nM protein was mixed with each redox-active reagent in a microcentrifuge tube. The mixture was then transferred into a polystyrene 96-well assay plate. Endpoint fluorescence measurements were obtained using a monochromator-based BioTek (Winooski, VT, USA) Synergy Mx Microplate Reader with excitation at 525 nm (20 nm bandwidth) and emission at 580 nm (20 nm bandwidth). For kinetics measurements, the protein at a concentration of 500 nM was first placed in the assay plate, and the initial fluorescence intensity was recorded. Subsequently, the respective redox reagents were added to each well, and subsequent fluorescence measurements were taken at specific time intervals. Metal specificity assays were performed following previously reported procedures [7]. To determine the molar mass of proteins, mass spectrometry analysis was performed using a Waters (Milford, MA, USA) SQD2 electrospray ionization mass spectrometer (ESI-MS). The protein was precipitated with methanol and chloroform, and then dissolved in ddH2O with 1% formic acid. The resulting solution was injected into the mass spectrometer, and the ESI peaks were deconvoluted using MagTran1.03 software to determine the molar mass of the protein.

## 3. Results

### 3.1. Optimization of the Expression of aY-cpGFP in Bacteria

Initially, we believed that the red fluorescence observed in aY-modified GFP or cpGFP was due to the additional oxidation of the GFP-like chromophore to form an RFP-like chromophore. However, some recent findings have disproven this hypothesis [38,43]. Instead, the new results indicate that the amino-derivatized GFP-like chromophore itself is sufficient for drastically red-shifted fluorescence. Therefore, we conclude that the presence of undesired green species observed in certain protein preparations is not due to incomplete conversion of the GFP-like chromophore to the RFP-like chromophore. Instead, we postulate that the misincorporation of Tyr by the engineered tRNA/aaRS pair and/or the instability of the aY-derived chromophore may be responsible for the occurrence of these undesired green species.

To begin our investigation, we optimized the expression conditions for aY-cpGFP. Our goal was to identify the most effective conditions that would promote the formation of the red-emitting product. In this study, we used a cpGFP scaffold derived from pnGFP-Ultra, which was initially created by introducing *p*-boronophenylalanine to the chromophore for the detection of peroxynitrite [40]. While the tyrosine-derived chromophore is formed in this cpGFP scaffold, the protein is known to be devoid of intrinsic redox sensitivity [40]. This characteristic is important as we planned to test the response of the resultant aY-cpGFP to ROS and RNS. It ensures that if any fluorescence response is observed, it is primarily due to the aY-derived chromophore. Additionally, the engineered termini of this cpGFP scaffold are strategically positioned close to the chromophore. The termini are structurally dynamic, allowing small molecules to enter and potentially interact or react with the chromophore.

**Figure 1:**
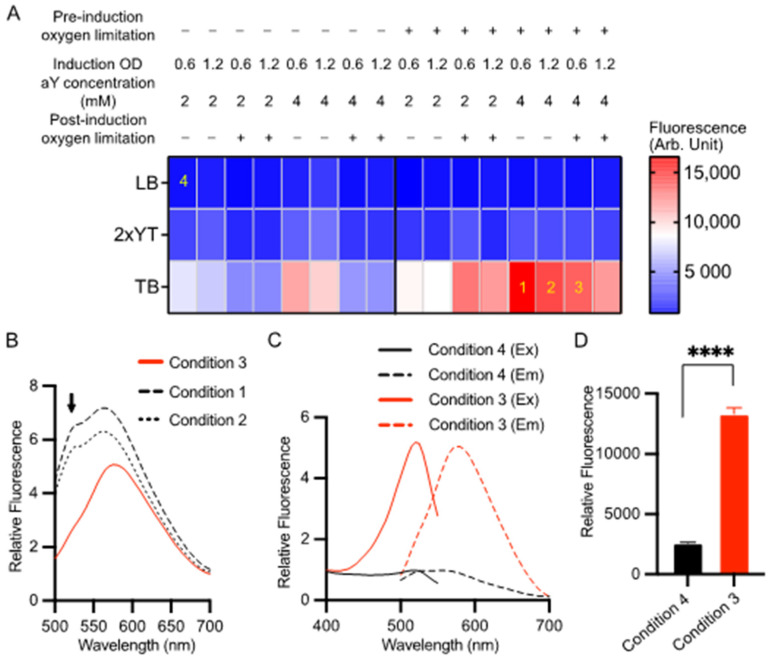
Optimization of the expression condition for aY-cpGFP in bacteria. (**A**) Screening of 48 conditions for the expression of aY-cpGFP in *E. coli* at 30 °C. The varied conditions included three types of culture media (LB, 2xYT, or TB), two aY concentrations (2 or 4 mM), the timing of bacterial culture induced with 0.2% l-arabinose (OD_600_ = 0.6 or 1.2), and whether to limit oxygen pre- or post-induction of protein expression. The red fluorescence of cell lysates was quantified for each expression condition, and the average intensity values were color-coded according to the provided calibration bar (N = 3 technical replicates). Four specific conditions labeled as 1–4 are referenced in other panels of this figure. (**B**) Fluorescence emission spectra of aY-cpGFP in bacterial lysates from conditions 1–3 depicted in panel A. (**C**,**D**) Normalized fluorescence excitation and emission spectra (**C**) and intensity values (**D**) of aY-cpGFP in bacterial lysate expressed under the optimized condition 3 (Red) compared to an un-optimized condition 4 (black). In panel (**D**), data are presented as mean ± SD of three technical replicates (**** *p* < 0.0001, two-tailed unpaired *t*-test).

Previously, we successfully expressed aY-sfGFP in sealed containers with a medium volume approximately equal to 1/5 of the container’s volume [38]. This approach, referred to as “oxygen limitation,” was implemented due to the sensitivity of aY to oxidation by molecular oxygen. Protein expression was induced using 0.2% L-arabinose at a temperature of 30 °C. In this study, we conducted a more comprehensive optimization of aY-cpGFP expression by assessing 48 different protein expression conditions (Figure 1A). We manipulated factors such as medium composition, oxygen availability, cell density at induction, and aY concentrations. Throughout the optimization process, we maintained consistent inducer concentrations (0.2% L-arabinose) and expression temperatures (30 °C). Our findings indicate that both increased medium nutrition levels and pre-induction oxygen limitation consistently resulted in higher protein expression levels (Figure 1A). Notably, the highest red fluorescence was observed when aY-cpGFP was expressed in TB medium with 4 mM aY, utilizing pre-induction oxygen limitation and inducing at OD_600_ = 0.6, while post-induction oxygen limitation was not implemented (referred to as condition 1 in Figure 1A). However, upon closer examination of the emission spectrum, it became apparent that there were also green-emitting species present (Figure 1B). We thus further examined the emission spectra of cell lysates from two additional conditions (Conditions 2 and 3 in Figure 1A) that exhibited strong red fluorescence. Among these conditions, post-induction oxygen limitation (Condition 3) yielded the best outcome with minimal undesired green emission. Consequently, we referred to this condition as “the optimized condition”, which involved using 4 mM aY, inducing at OD600 = 0.6, and implementing both pre- and post-induction oxygen limitation. By employing this optimized expression condition, we could consistently obtain the pure aY-cpGFP proteins with a single excitation (λ_ex_ = 521 nm) and emission (λ_em_ = 590 nm) peaks (Figure 1C). Moreover, the optimized expression condition has resulted in a satisfactory protein production level, as evidenced by the remarkably increased fluorescence intensity (Figure 1D). Following the Ni-NTA affinity purification, the protein underwent size-exclusion chromatography, which indicated a predominantly monomeric nature (Supplementary Materials, Figure S1). The successful incorporation of aY into this protein was further verified through mass spectrometry analysis (Figure S2).

### 3.2. Brightness Improvement through Protein Engineering

**Figure 2:**
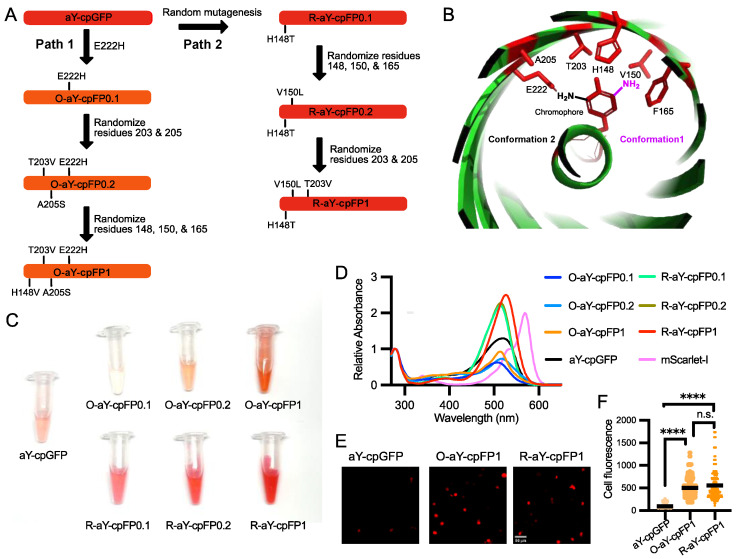
Engineering and initial characterization of aY-derived cpFPs. (**A**) Schematic illustration of the process used to engineer O-aY-cpFP1 and R-aY-cpFP1 from aY-cpGFP. (**B**) Illustration of the local environment of the aY-cpGFP chromophore. This graph was rendered based on the Protein Data Bank (PDB) entry 5F9G. The chromophore-forming tyrosine and the amino acid residues surrounding the chromophore are colored red. Two possible conformations of the amino-derivatized chromophore are shown in black and magenta, respectively. (**C**) Images of aY-charged cpFPs prepared from the same amounts of *E. coli*. (**D**) Absorbance spectra of aY-cpGFP, O-aY-cpFPs, R-aY-cpFPs, and mScarlet-I normalized at the wavelength of 280 nm. (**E**) Representative images of aY-cpGFP, O-aY-cpFP1, and R-aY-cpFP1 expressed in HEK 293T cells. Scale bar, 50 µm. (**F**) Fluorescence intensity quantification of HEK293T cells expressing aY-cpGFP, O-aY-cpFP1, or R-aY-cpFP1. Data were analyzed with an ordinary one-way ANOVA followed by Tukey’s multiple comparison test (**** *p* < 0.0001; n.s., not significant; *p* > 0.05).

**Figure 3:**
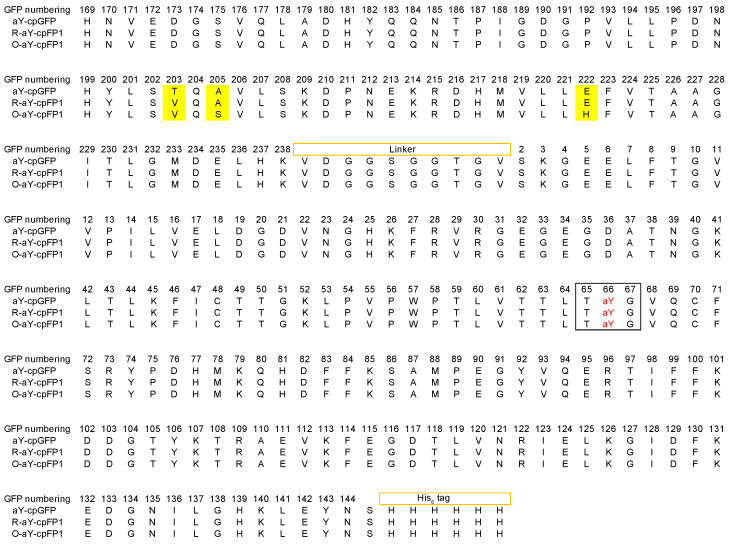
Sequence alignment of aY-cpGFP, R-aY-cpFP1, and O-aY-cpFP1. Mutations are highlighted in yellow. In the black box are the amino acid residues responsible for chromophore formation. The residues are numbered following the wild-type GFP.

The molar excitation coefficient and quantum yield of the carefully prepared aY-cpGFP were determined to be 48.7 mM^−1^ cm^−1^ and 0.14 (Table 1), respectively. To further enhance its brightness, we proceeded with protein engineering (Figure 2A). In our recent study, we demonstrated that introducing a single E222H mutation near the amino functional group of the aY-modified chromophore (Figure 2B) drastically increased the quantum yield of aY-sfGFP [38]. Building upon this finding, we incorporated the same mutation into aY-cpGFP. As a result, we obtained a fluorescent protein with a faint orange color, exhibiting excitation and emission maxima at 512 and 571 nm, respectively (Figure 2C and Figure S3). We named this protein O-aY-cpFP0.1. Although O-aY-cpFP0.1 displayed a nearly two-fold improvement in quantum yield, it was expressed at a considerably lower level compared to the original protein (Table 1 and Figure S1). Next, we proceeded to choose two additional neighboring residues (residues 203 and 205, following the numbering of wild-type GFP) and conducted site-directed saturation mutagenesis. As a result, we obtained O-aY-cpFP0.2, which includes T203V and A205S mutations. Additionally, we performed mutagenesis at residues 148, 150, and 165, which were predicted to be in proximity to the amino functional group of the aY-modified chromophore in an alternative conformation (as shown in Figure 2B). This led us to discover a mutant with an additional H148V mutation. We designated this mutant as O-aY-cpFP1, which exhibited excitation and emission maxima at 513 and 577 nm, respectively (Figure 3 and Figure S3). The molecular brightness of O-aY-cpFP1 was doubled from aY-cpGFP, due to the increased quantum yield (Table 1). The proteins of O-aY-cpFPs from Ni-NTA affinity purification were injected into a size-exclusion column, and we predominantly observed peaks corresponding to monomeric proteins (Figure S1). However, we also detected minor peaks at the elution volume of approximately 50 mL, indicating a much larger molecular size. This suggests the presence of some instances of oligomerization or aggregation of O-aY-cpFPs.

In an alternative approach, we initially conducted random mutagenesis on aY-cpGFP (Figure 2A). Following the screening of approximately 20,000 colonies, we identified R-aY-cpFP0.1, which featured a single H148T mutation (based on the numbering of wild-type GFP) in close proximity to the chromophore. R-aY-cpFP0.1 exhibited a red color and a largely increased extinction coefficient (Table 1). Subsequently, we performed site-directed saturation mutagenesis simultaneously on several residues surrounding the chromophore (specifically residues 148, 150, and 165). Through screening this library, we discovered R-aY-cpFP0.2, which contained an additional V150L mutation. Finally, we conducted mutagenesis on residues 203 and 205, leading to the identification of R-aY-cpFP1, which featured an additional T203V mutation. R-aY-cpFP1 exhibited a molecular brightness approximately 2.8 times higher than that of aY-cpGFP, primarily due to its increased extinction coefficient. Additionally, R-aY-cpFP1 demonstrated notably higher expression levels, resulting in a remarkable overall 13-fold increase in apparent brightness in *E. coli* lysate (Table 1 and Figure S1). Size-exclusion chromatography analysis demonstrated that R-aY-cpFPs primarily exist as monomers, indicating a decreased tendency for aggregation compared to their O-aY-cpFP counterparts.

We made an additional effort to combine the advantageous mutations from both O-aY-cpFP1 and R-aY-cpFP1, but unfortunately, our attempt was unsuccessful. Introducing A205S and E222H into R-aY-cpFP1 did not lead to an increase in fluorescence intensity. Furthermore, conducting site-directed saturation mutagenesis at residue 222 of R-aY-cpFP1 did not produce any brighter mutants.

The successful incorporation of aY into O-aY-cpFP1 and R-aY-cpFP1 proteins was confirmed through mass spectrometry analysis using purified proteins (Figures S4 and S5). In addition, we performed fluorescence lifetime measurements to gain further insight. O-aY-cpFPs exhibited longer fluorescence lifetimes, while the lifetimes of R-aY-cpFPs were comparable to that of aY-cpGFP (Table 1). This distinction provides additional evidence that different mechanisms are responsible for the improvement in brightness within these two mutant series. Additionally, we investigated the pH sensitivity of these proteins. O-aY-cpFPs displayed higher apparent *p*K_a_ values compared to aY-cpGFP, whereas R-aY-cpFPs exhibited lower *p*K_a_ values (Table 1 and Figure S6).

O-aY-cpFP1 and R-aY-cpFP1 emerged as the most promising mutants from the protein engineering process. To investigate if the brightness improvement could be extended to a mammalian cell setting, we expressed O-aY-cpFP1, R-aY-cpFP1, and aY-cpGFP in HEK293T cells (Figure 2D). Notably, both O-aY-cpFP1 and R-aY-cpFP1 exhibited significantly enhanced brightness compared to aY-cpGFP (Figure 2E). O-aY-cpFP1 and R-aY-cpFP1 gave comparable brightness in mammalian cells (Figure 2E), although the apparent brightness of R-aY-cpFP1 in *E. coli* was much higher (Table 1).

### 3.3. Metal Ion and Redox Responses of R-aY-cpFP1

We next selected R-aY-cpFP1 for further characterization, due to its red-shifted fluorescence spectra, higher molecular brightness, and significantly enhanced brightness in *E. coli* (Figure S3 and Table 1). Our specific area of interest for characterization centered around investigating the sensitivity of R-aY-cpFP1 fluorescence to metal ions, ROS, and RNS.

**Figure 4:**
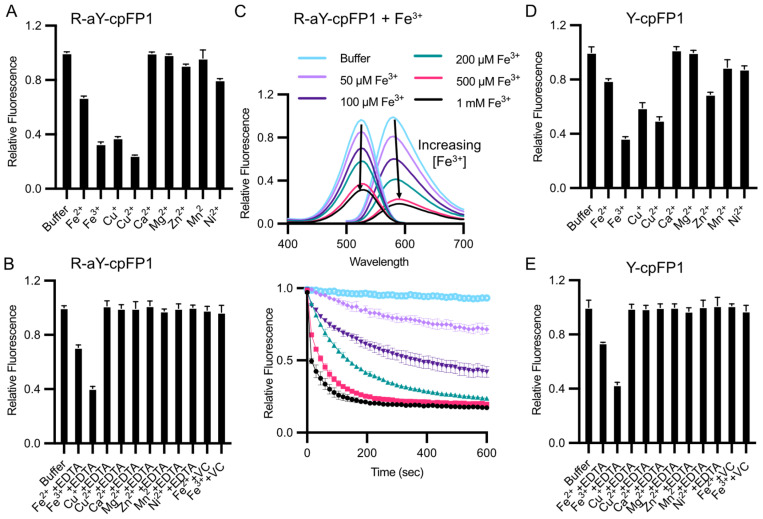
Sensitivity of R-aY-cpFP1 and Y-cpFP1 to metal ions. (**A**) Normalized fluorescence intensity of 500 nM R-aY- cpFP1 treated with various metal ions (100 μM). (**B**) Normalized fluorescence intensity of 500 nM R-aY-cpFP1 first treated with the indicated metal ions (100 μM), followed by 10 mM EDTA or 50 mM vitamin C (VC) for another 15 min. (**C**) Top panel: Fluorescence excitation and emission spectra of 500 nM R-aY-cpFP1 after 2 min treatment with Fe^3+^ at the indicated concentrations. Bottom panel: Time-lapse traces (F/F_0_) of fluorescence intensity of 500 nM R-aY-cpFP1 treated with Fe^3+^. (**D**) Normalized fluorescence intensity of 500 nM Y-cpFP1 treated with various metal ions (100 μM). (**E**) Normalized fluorescence intensity of 500 nM Y-cpFP1 first treated with the indicated metal ions (100 μM), followed by 10 mM EDTA or 50 mM vitamin C (VC) for another 15 min. The top panel in (**C**) presents one trial of representative results. Other panels present mean ± SD from three technical replicates.

Among common metal ions at a concentration of 100 μM, the fluorescence of R-aY-cpFP1 exhibited a decrease in response to Fe^2+^, Fe^3+^, and Cu^2+^ (Figure 4A). Additionally, Zn^2+^ and Ni^2+^ caused a minor decrease in fluorescence. However, when the metal chelator ethylenediaminetetraacetic acid (EDTA) was subsequently added to R-aY-cpFP1 that was pre-treated with the metals, the metal-induced fluorescence changes were effectively reversed, except in the case of Fe^2+^ or Fe^3+^ (Figure 4B). Notably, the fluorescence quenching induced by Fe^3+^ was found to be both dose- and time-dependent (Figure 4C). Furthermore, when vitamin C (VC, also known as sodium ascorbate) was introduced to R-aY-cpFP1 that was pre-treated with Fe^2+^ or Fe^3+^, the metal-induced fluorescence changes were reversed (Figure 4B). Since Fe^2+^ reagents often have Fe^3+^ impurities due to oxidation by molecular oxygen and vitamin C can convert Fe^3+^ to Fe^2+^, the initially observed Fe^2+^-induced fluorescence quenching (Figure 4A) was likely an artifact. Also, the results suggest that Fe^3+^ either forms a highly stable interaction with the protein that cannot be reversed by EDTA, or it undergoes a redox reaction with the protein that necessitates the presence of the reductant vitamin C for reversion.

Subsequently, we prepared Y-cpFP1, which is nearly identical to R-aY-cpFP1 except for the presence of a tyrosine-derived chromophore. Y-cpFP1 lacks the extra amino group in its chromophore (Figure 2B). Interestingly, we observed a similar pattern of response to metal ions with Y-cpFP1, albeit with a few minor differences in terms of the magnitude of fluorescence quenching (Figure 4D). Additionally, the responses of metal-ion-pretreated Y-cpFP1 to EDTA and vitamin C were comparable to those observed with R-aY-cpFP1 (Figure 4E). These findings indicate that the metal sensitivity of R-aY-cpFP1 closely resembles that of Y-cpFP1, with only slight variations.

**Figure 5:**
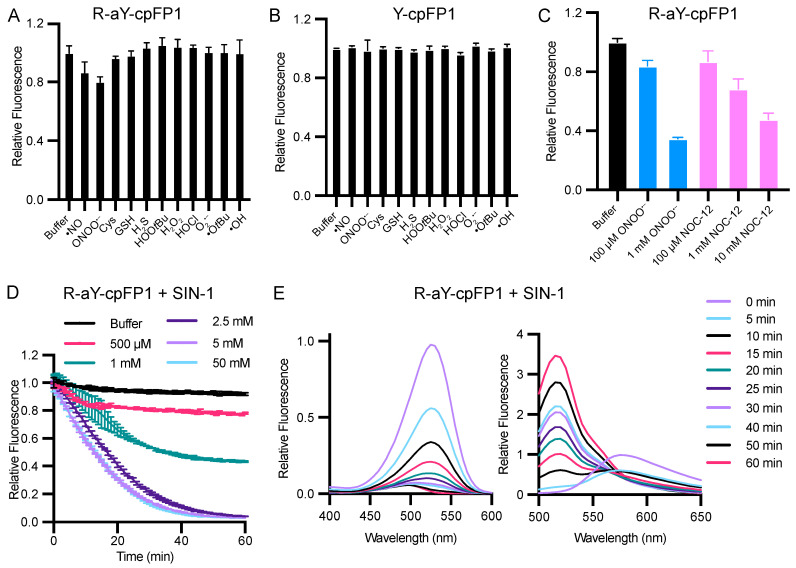
Redox sensitivity of R-aY-cpFP1 and Y-cpFP1. (**A**,**B**) Normalized fluorescence intensity of R-aY-cpFP1 or Y-cpFP1 (500 nM) treated with various oxidants or reductants (from left to right: buffer, 100 μM NOC-12 (•NO donor), 100 μM ONOONa, 5 mM cysteine, 5 mM GSH, 100 μM neutralized NaHS, 100 μM HOO*t*Bu, 100 μM H_2_O_2_, 100 μM neutralized NaOCl, 100 μM KO_2_, 1 mM Fe^2+^ + 100 μM HOO*t*Bu + 10 mM EDTA(•O*t*Bu); 1 mM Fe^2+^ + 100 μM H_2_O_2_ + 10 mM EDTA (•OH)). (**C**) Normalized fluorescence intensity of R-aY-cpFP1 (500 nM) treated with the indicated concentrations of ONOO^−^ or NOC-12. (**D**) Time-lapse responses of R-aY-cpFP1 (500 nM) to the indicated concentrations of SIN-1. (**E**) Normalized excitation (left) and emission (right) spectra of R-aY-cpFP1 (500 nM) treated with 2.5 mM SIN-1 at different time points. Panel E presents one trial of representative results. Other panels present mean ± SD from three technical replicates.

In addition, we investigated the redox sensitivity of R-aY-cpFP1 by subjecting the protein to various ROS, RNS, and reductants. Among the molecules tested, R-aY-cpFP1 exhibited a significant decrease in fluorescence in response to peroxynitrite (ONOO^−^) and nitric oxide (•NO) (Figure 5A). Conversely, Y-cpFP1 did not demonstrate any response to the tested ROS, RNS, and reductants (Figure 5B). The responses of R-aY-cpFP1 to peroxynitrite and nitric oxide were found to be dose-dependent (Figure 5C). Specifically, at 1 mM concentration, peroxynitrite caused a fluorescence reduction of approximately 65%, while 10 mM NOC-12, a nitric oxide donor, resulted in a 52% fluorescence decrease.

In a neutral aqueous solution, peroxynitrite undergoes a rapid decomposition [44]. To further investigate the reaction between R-aY-cpFP1 and peroxynitrite, we used SIN-1 (3-morpholino-sydnonimine), a molecule that slowly releases peroxynitrite by simultaneously producing nitric oxide and superoxide. Time- and dose-dependent fluorescence quenching was observed (Figure 5D), confirming the reaction between R-aY-cpFP1 and peroxynitrite. In the presence of 2.5 mM SIN-1, equivalent to a peroxynitrite production rate of approximately 35 μM per minute [45], the red fluorescence of R-aY-cpFP1 almost completely diminished within 60 min (Figure 5D). Simultaneously, fluorescence excitation and emission profiles similar to those of GFP and Y-cpFP1 appeared (Figure 5E and Figure S7). This indicates that SIN-1 converted the red-emitting protein into a green-emitting protein. We used mass spectrometry to further analyze the reaction products. The results support the notion that peroxynitrite can induce deamination of the aY-derived chromophore, converting it into a tyrosine-derived chromophore (Figure S8). Additionally, a peak corresponding to the nitration of the protein by peroxynitrite was also observed using mass spectrometry.

### 3.4. Expression of R-aY-cpFP1 in Mammalian Cells

**Figure 6:**
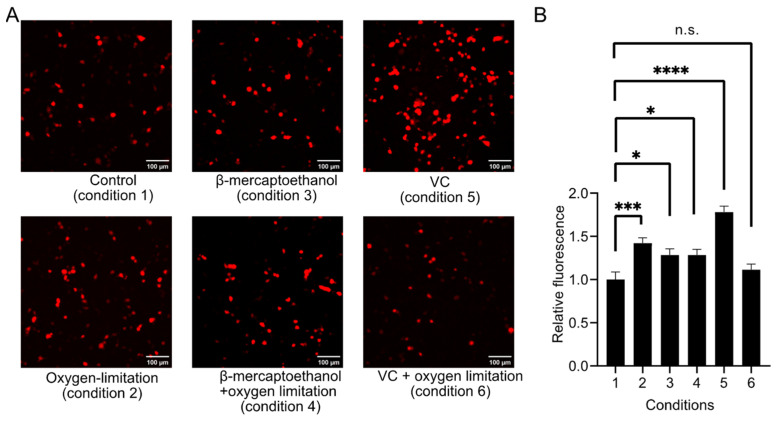
Optimization of the expression condition for R-aY-cpFP1 in HEK293T cells. (**A**) Representative images of R-aY-cpFP1 expressed in HEK 293T cells under various conditions. Scale bar, 100 µm. The varied conditions included the addition of reductant (0.5 mM β-mercaptoethanol) or antioxidant (20 μM VC), and whether to limit oxygen by sealing the culture dish. (**B**) Fluorescence intensity quantification of HEK 293T cells expressing R-aY-cpFP1 under various conditions. Data were analyzed with an ordinary one-way ANOVA followed by Dunnett’s multiple comparison test (* *p* < 0.05; *** *p* < 0.001; **** *p* < 0.0001; n.s., not significant; *p* > 0.05).

After knowing the sensitivity of aY to oxidation and the reactivity of R-aY-cpFP1 with RNS, we proceeded to evaluate the expression of R-aY-cpFP1 in HEK293T cells by reducing oxidation. We implemented six different cell culture conditions following the addition of aY to the culture medium (Figure 6A). Specifically, we examined the red fluorescence of R-aY-cpFP1 in HEK293T cells under various conditions, including sealing the culture dishes or adding reductants like β-mercaptoethanol and VC to the culture media. Interestingly, we observed significant enhancements in R-aY-cpFP1 expression under four of the tested conditions (Figure 6B). Both oxygen limitation and β-mercaptoethanol addition independently increased R-aY-cpFP1 expression, although there was no additive effect when combined. The most substantial enhancement was observed with the addition of VC alone. When VC was added along with oxygen limitation, there was no significant improvement.

## 4. Discussion

The incorporation of aY into GFP-like chromophores has been proven convenient for achieving red-shifted fluorescence. However, the practical implementation of this method has resulted in inconsistent outcomes. In this study, we focused on aY-cpGFP as a model protein and aimed to optimize its expression to obtain the high-yield and high-fidelity production of the red-emitting protein. Through our investigations, we identified an optimized condition that significantly improved the reliability and consistency of the results. One crucial factor we found was the use of high-nutrition culture media during the expression process. TB medium provides a considerably higher nutrient content in the form of yeast extract and tryptone compared to LB or 2xYT medium. Additionally, TB includes phosphate-based buffering components, which contribute to maintaining the pH of the culture. These favorable conditions enable bacteria to achieve a significantly higher density and facilitate the expression of a larger quantity of proteins. Additionally, we implemented a strategy to limit oxygen availability throughout the entire expression process. This restriction of oxygen helped to maintain the stability and integrity of the aY amino acid and its derived protein, resulting in improved fidelity and reduced occurrence of undesired green-emitting species. It is important to mention that our experiment did not entirely eliminate oxygen, which is necessary for both cell growth and chromophore maturation. We utilized sealed containers where the medium occupied approximately 1/5 of the container’s volume, while the remaining 4/5 consisted of air before sealing. This specific volume ratio played a significant role in achieving optimal outcomes.

In addition, we re-determined the photophysical properties of aY-cpGFP using the pure protein. Moreover, we explored the impact of mutating specific amino acid residues surrounding the chromophore on the brightness of aY-cpGFP. We successfully obtained brighter mutants by introducing mutations to a small number of amino acid residues. The resulting mutants exhibited photophysical properties that were comparable to those of commonly used cpRFP-based biosensors [8,46,47]. Interestingly, starting with mutagenesis targeting residues on one side of the chromophore (E222, T203, and S205) or the other side (H148, V150, and F165) resulted in the creation of two distinct sets of aY-cpFPs, exhibiting quite different emission wavelengths and some other biophysical properties. This finding provides valuable insights into the structural and functional aspects of aY-cpFPs and opens up possibilities for further optimization.

We further tested the stability of the resultant R-aY-cpFP1 in the presence of common metal ions. Previous studies have reported that certain metal ions can form complexes with or even catalyze the oxidation of 2-aminophenol, which is the functional group within aY [48,49,50,51,52]. Surprisingly, our results indicate that aY, when incorporated into the folded R-aY-cpFP1 protein, displayed a good level of stability. We observed that metal-induced fluorescence quenching of R-aY-cpFP1 was generally reversible, and the response pattern was not remarkably different from that of Y-cpFP1, which is a similar protein but with a tyrosine-derived chromophore.

Furthermore, we tested the responses of R-aY-cpFP1 to ROS and RNS. R-aY-cpFP1 exhibited responses to peroxynitrite and nitric oxide at relatively high but still physiologically relevant concentrations. Since Y-cpFP1 did not respond under these conditions, the responses of R-aY-cpFP1 to RNS were likely due to the aY-derived chromophore. The fluorescence spectra of R-aY-cpFP1 treated with SIN-1 resembled those of Y-cpFP1. Mass spectrometry detected protein nitration, in addition to a major peak corresponding to the loss of an amino group, suggesting that peroxynitrite could remove the amino functional group from the aY-derived chromophore. This finding corroborates a previous report on the removal of the amine group of a 2-aminophenol-containing molecule by nitric oxide in the presence of molecular oxygen [53].

Moreover, we successfully expressed R-aY-cpFP1 in mammalian cells. Through our experiments, we identified that oxygen limitation and the addition of β-mercaptoethanol or VC enhanced the expression of R-aY-cpFP1. Among these factors, the most substantial improvement in expression was observed when 20 μM VC was added to the culture medium. This finding offers valuable insights for further optimization of cell culture conditions to enhance the robustness of aY-derived, red-shifted biosensors in mammalian cells.

We would also like to acknowledge the limitations of our study as we still cannot conclude the reasons behind the presence of undesired green fluorescence species. Although it appeared that the aY-derived chromophore could potentially be converted to a tyrosine-derived chromophore by RNS, the concentrations used in our in vitro assays were relatively high. Moreover, *E. coli* cells are not known to naturally generate nitric oxide or peroxynitrite unless specific circumstances are met [54,55]. Therefore, it is unlikely that the green-emitting species, at least during *E. coli* expression, were caused by the RNS-induced conversion of the aY-derived chromophore to a tyrosine-derived chromophore. On the other hand, the oxidation of aY could still occur in the early steps of protein synthesis. Since aY starts as a free amino acid and is subsequently incorporated into an aminoacyl-tRNA and an unfolded peptide, its reactivity under these conditions is likely different from its behavior when it is on the chromophore of the fully folded protein. We have observed the instability of the aY amino acid, as it gradually darkens in the culture media. The relative availability of aY and tyrosine would significantly influence the specificity of aY incorporation because oxidation-induced loss of aY may result in the potential mis-incorporation of tyrosine by the introduced tRNA and aaRS pair. It is also possible that cells possess alternative pathways to convert aY to tyrosine. Notably, 3-nitrotyrosine is an oxidative post-translational modification of tyrosine in proteins within living organisms. The reversibility of 3-nitrotyrosine is still a topic of debate, but several studies have suggested the enzymatic reversibility of 3-nitrotyrosine [56,57,58]. Therefore, we cannot exclude the possibility of the existence of enzymatic conversion of aY to Y in proteins.

Another notable finding is that R-aY-cpFP1 exhibited limited reactivity towards metal ions and ROS. This suggests that incorporating aY remains a viable approach to red-shift cpGFP-based biosensors. Further research is necessary to have a better mechanistic understanding and refine the approaches for even greater robustness.

## 5. Conclusions

In this study, we used aY-cpGFP as a model protein to achieve red-shifted fluorescence by introducing the aY amino acid to the chromophores of cpGFP. Through an extensive process, we successfully identified an optimized expression condition that involved rich culture media and oxygen restriction. This optimized condition enabled us to achieve the high-yield and high-purity production of the red-shifted protein. Additionally, we employed protein engineering techniques to develop two variants of aY-cpGFP with enhanced brightness by introducing specific amino acid mutations in the vicinity of the chromophore. Furthermore, we investigated the responsiveness of an aY-cpGFP mutant to common metal ions, as well as ROS and RNS. While the incorporation of aY into cpGFP had a minimal impact on metal ion reactivity, it increased the protein’s sensitivity to RNS. Moreover, when the aY-cpGFP mutant was expressed in mammalian cells, the presence of reductants in the culture media also enhanced the production of red-emitting products. Our study demonstrates that optimizing expression conditions to promote a reduced cellular state proved to be effective in producing the desired red-emitting product in both *E. coli* and mammalian cells. Additionally, we found that the photophysical properties of the converted aY-derived protein could be further improved by introducing mutations to a small number of residues near the chromophore. Considering previous successful research involving the fusion of cpGFP with various sensing domains to create diverse fluorescent biosensors, the methodology presented in this paper for enhancing aY-cpGFPs can be applied to optimize aY-cpGFP-based biosensors. Therefore, this study serves as a valuable guideline for the development and application of aY-based red fluorescent biosensors.

## Figures and Tables

**Table 1:** Photophysical properties of the indicated proteins.

Name	λ_ex_ (nm)	λ_em_ (nm)	Bacterial Lysate Brightness	*ε* (mM^−1^ cm^−1^)	*ϕ*	Molecular Brightness ^1^	*τ* (ns)	*p*K_a_ ^2^
aY-cpGFP	521	590	100%	48.7	0.14	6.7	1.0	6.8
O-aY-cpFP0.1	512	571	45%	39.1	0.26	10.1	2.7	7.2
O-aY-cpFP0.2	515	577	80%	46.6	0.21	9.9	2.6	7.4
O-aY-cpFP1	513	577	139%	39.2	0.29	11.5	2.6	7.0
R-aY-cpFP0.1	515	590	561%	114.7	0.16	18.6	1.2	6.3
R-aY-cpFP0.2	516	589	748%	118.8	0.16	19.0	1.2	6.3
R-aY-cpFP1	528	580	1312%	124.4	0.15	18.7	1.1	6.5

^1^ Defined as the product of *ε* (extinction coefficient) and *ϕ* (quantum yield). ^2^ pH when the fluorescence brightness is quenched by a half.

## Data Availability

The original data are available upon request from the corresponding author.

## Supplementary Materials

The following supporting information can be downloaded at: https://www.mdpi.com/article/10.3390/bios14010054/s1, Figure S1: Size-exclusion chromatography (SEC) elution profiles of different mutants; Figure S2: Electrospray ionization mass spectrometry (ESI-MS) analysis of intact aY-cpGFP protein (with a C-terminal His6 tag) purified from *E. coli*; Figure S3: Fluorescence spectra of different aY-cpFP mutants; Figure S4: ESI-MS analysis of intact R-aY-cpFP1 protein (with a C-terminal His6 tag) purified from *E. coli*; Figure S5: ESI-MS analysis of intact O-aY-cpFP1 protein (with a C-terminal His6 tag) purified from *E. coli*; Figure S6: pH dependency of the fluorescence of different aY-cpFP mutants; Figure S7: Overlay of fluorescence excitation (A) and emission (B) spectra of R-aY-cpFP1 (magenta), SIN-1-treated R-aY-cpFP1 (orange), and Y-cpFP1 (green); Figure S8: ESI-MS analysis of intact R-aY-cpFP1 protein (with a C-terminal His6 tag) after reaction with peroxynitrite; Table S1: List of oligos used in this study.
