# Supplementary material for: Engineering and Characterization of 3-Aminotyrosine-Derived Red Fluorescent Variants of Circularly Permutated Green Fluorescent Protein
Source: Biosensors (Basel). 2024 Jan 20;14(1):54. doi: 10.3390/bios14010054 (PMC10813706; doi:10.3390/bios14010054)
Supplement: Supplementary file 1 [file biosensors-14-00054-s001.zip › biosensors-2790208-supplementary.pdf]

biosensors

MDPI

Supplementary Materials

Engineering and Characterization of 3-Aminotyrosine-Derived Red Fluorescent Variants of Circularly Permutated Green Fluorescent Protein

Hao Zhang [1,2], Xiaodong Tian [1,3], Jing Zhang [1,3] and Hui-wang Ai [1,2,3,4,*]

1 Center for Membrane and Cell Physiology, University of Virginia, Charlottesville, VA 22908, USA; hz5qd@virginia.edu (H.Z.); xt3eg@virginia.edu (X.T.); jz4m@virginia.edu (J.Z.)
2 Department of Chemistry, University of Virginia, Charlottesville, VA 22904, USA
3 Department of Molecular Physiology and Biological Physics, University of Virginia, Charlottesville, VA 22908, USA
4 The UVA Comprehensive Cancer Center, University of Virginia, Charlottesville, VA 22908, USA
* Correspondence: huiwang.ai@virginia.edu

Table S1. List of oligos used in this study.

Primer name	Sequence (5′ → 3′)
E222H-f	5′-CATGGTCCTGCTGCACTTCGTGACCGCC-3′
E222H-r	5′-GGCGGTCACGAAGTGCAGCAGGACCATG-3′
203205X-f	5′-CACTACCTGAGCNNKCAGNNKGTGCTGAGCAAAG-3′
203205X-r	5′-CTTTGCTCAGCACMNNCTGMNNGCTCAGGTAGTG-3′
NNKx3-f	5′-CCGACAAGCAGAAGAACGGCATCAAGGCGAACNN-KCAGATCCGCCACAACG-3′
NNKx3-r	5′-GTTCTTCTGCTTGTCGGCGGTGATATAMNNCTTMNNGCTGTT-GTACTTCTTGC-3′
E222X-f	5′-CATGGTCCTGCTGNNKTTCGTGACCGCC-3′
E222X-r	5′-GGCGGTCACGAAMNNCAGCAGGACCATG-3′
148T150L-f	5′-GAAGTACAACAGCACCAAGCTCTATATCACCGCC-3′
148T150L-r	5′-GGCGGTGATATAGAGCTTGGTGCTGTTGTACTTC-3′
pMAH-r	5′-GGGTTTAAACGGGCCCTTGGTCACGAGTTGTACTCCAGCTTG-3′
pMAH-f	5′-CAACTGCACGGAAGCTTGCCACCATGGGCTCGAGCAAGAAG-3′
pBAD-f	5′-ATTAACCATGGGCTCGAG-3′
pBAD-r	5′-GCCAAAACAGCCAAGCTT-3′

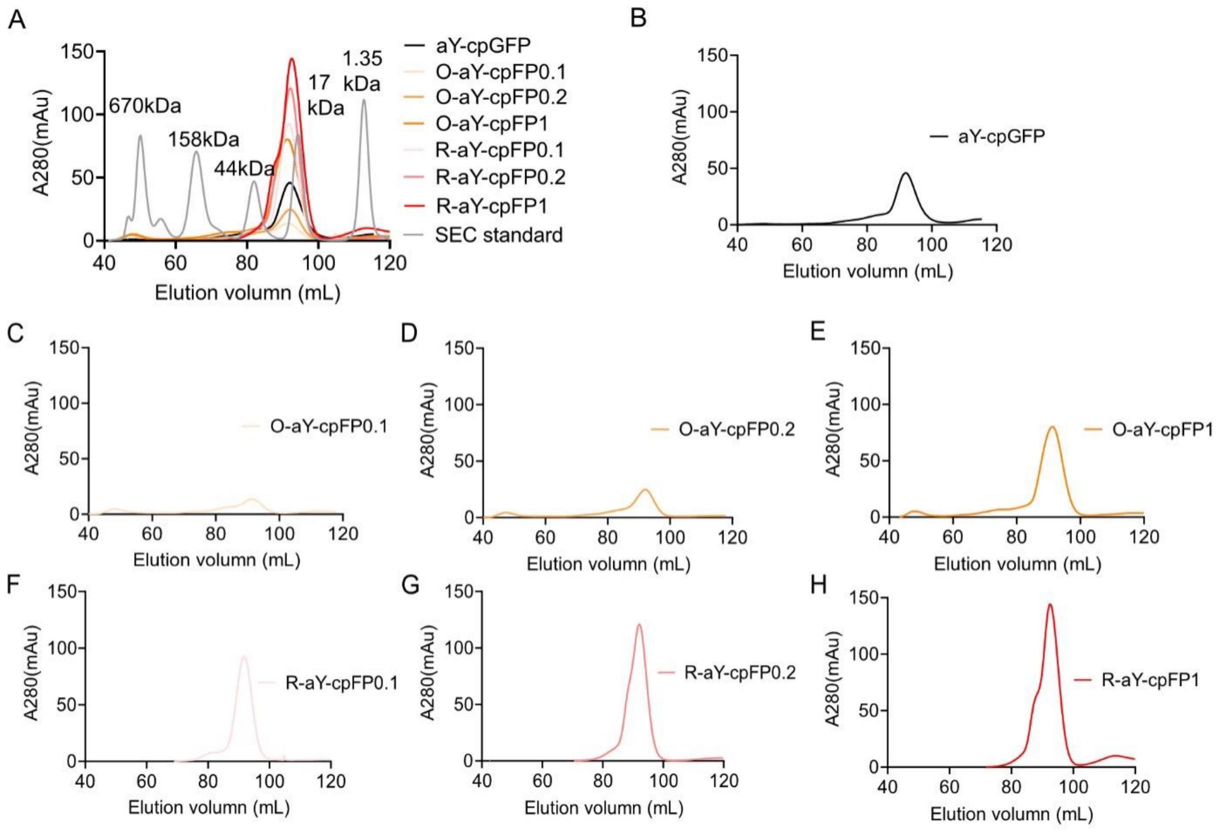

Figure S1. Size-exclusion chromatography (SEC) elution profiles of different mutants. The absorbance detection was at 280 nm. (**A**) Overlay of SEC standards and all mutants expressed from the same amount of culture media, (**B-G**) The SEC elution profiles of individual mutants.

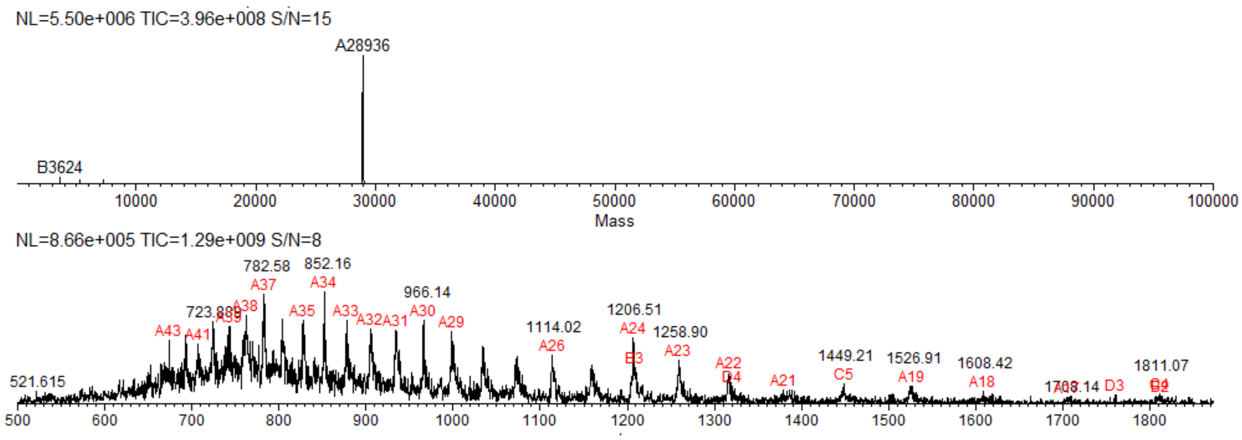

Figure S2. Electrospray ionization mass spectrometry (ESI-MS) analysis of intact aY-cpGFP protein (with a C-terminal His6 tag) purified from *E. coli*. The observed mass matched the calculated mass (calculated mass: 28936 Da). No peak corresponding to cpGFP with tyrosine at residue 66 (calculated mass: 28921 Da) was observed.

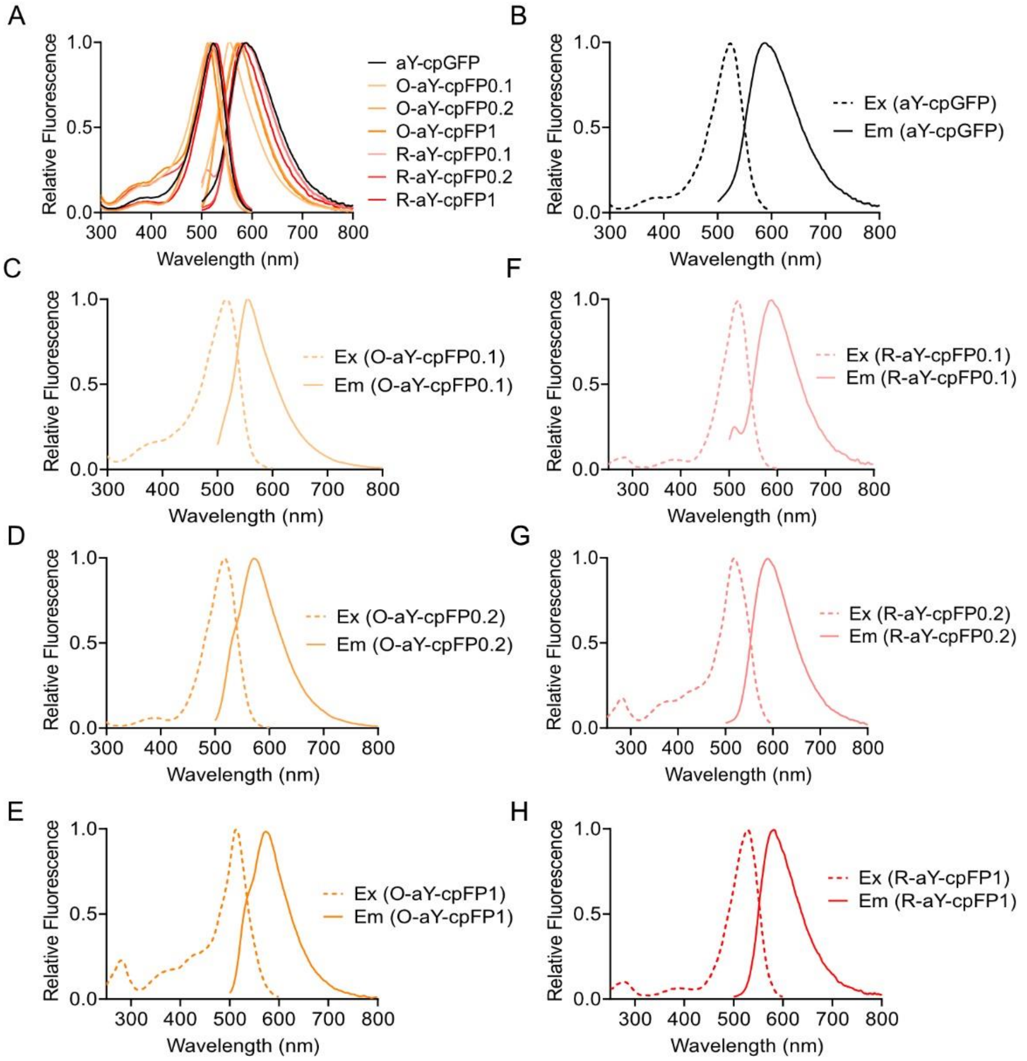

Figure S3. Fluorescence spectra of different aY-cpFP mutants. (**A**) Overlay of the fluorescence excitation and emission spectra. (**B-G**) Fluorescence excitation and emission spectra of individual mutants.

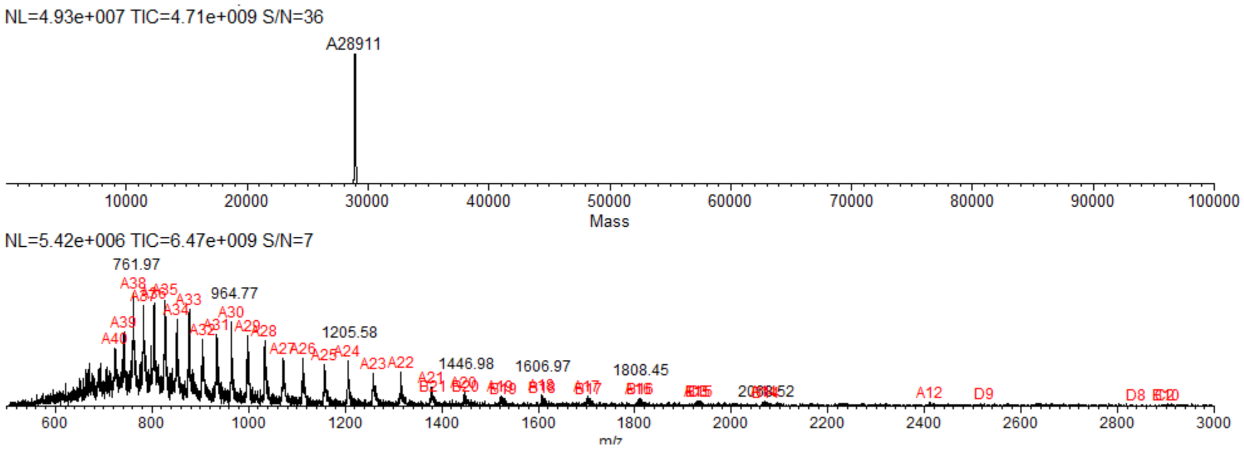

Figure S4. ESI-MS analysis of intact R-aY-cpFP1 protein (with a C-terminal His6 tag) purified from *E. coli*. The observed mass matched the calculated mass (calculated mass: 28912 Da).

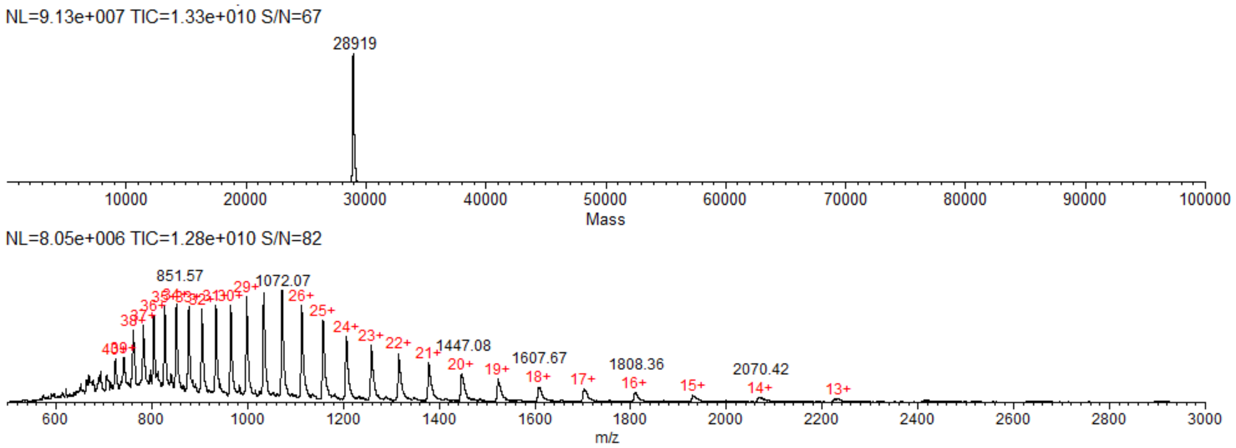

Figure S5. ESI-MS analysis of intact O-aY-cpFP1 protein (with a C-terminal His6 tag) purified from *E. coli*. The observed mass matched the calculated mass (calculated mass: 28919 Da).

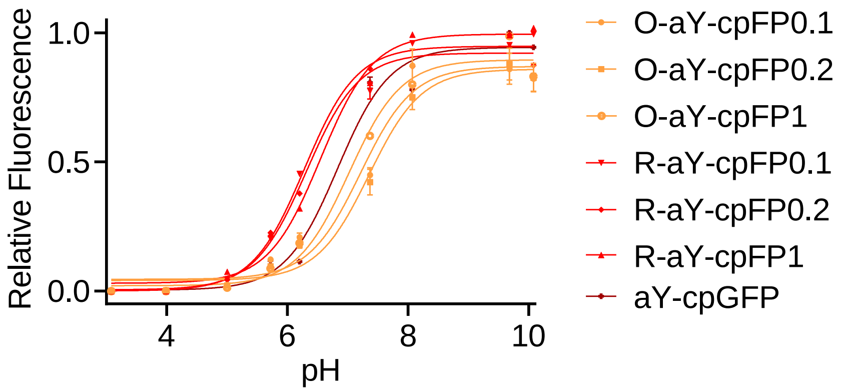

Figure S6. pH dependency of the fluorescence of different aY-cpFP mutants. Data are presented as mean ± SEM of three technical replicates. Lines are the fitting of the data with the Hill equation, and apparent pK_a values (defined as the pH causing 50% of the overall fluorescence intensity change) are derived and presented in Table 1.

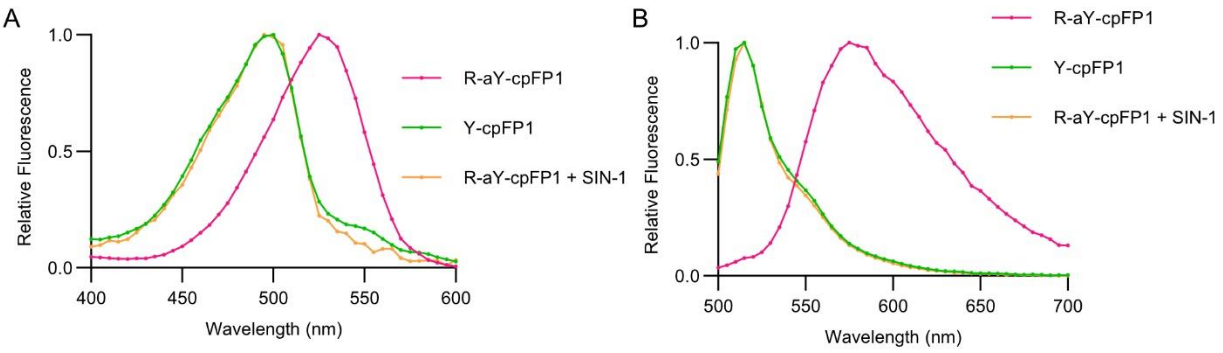

Figure S7. Overlay of fluorescence excitation (**A**) and emission (**B**) spectra of R-aY-cpFP1 (magenta), SIN-1-treated R-aY-cpFP1 (orange), and Y-cpFP1 (green). Y-cpFP1 has the same protein sequence as R-aY-cpFP1 except that Y-cpFP1 has a tyrosine-derived chromophore.

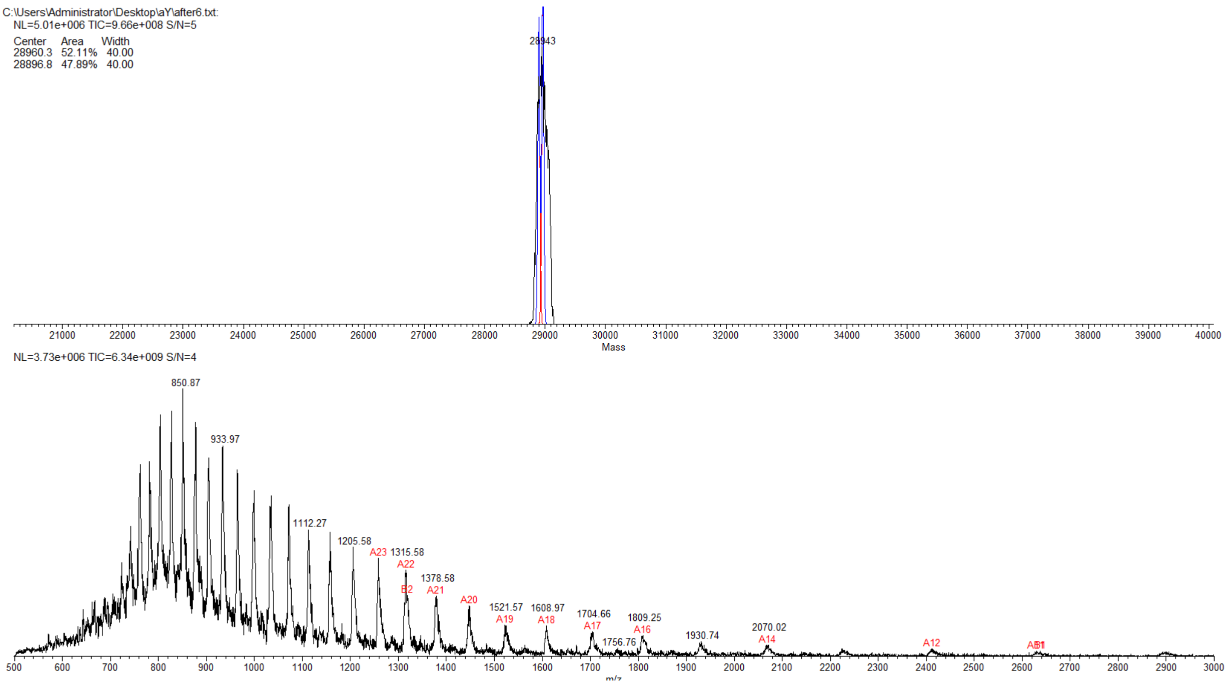

Figure S8. ESI-MS analysis of intact R-aY-cpFP1 protein (with a C-terminal His6 tag) after reaction with peroxynitrite. The observed mass showed two major peaks after deconvolution. One of the masses matched with the calculated mass of Y-cpFP1 (calculated mass: 28896 Da), suggesting a deamination of R-aY-cpFP1. The other mass matched with the calculated mass of nitrated R-aY-cpFP1 (calculated mass: 28960 Da), suggesting the nitration of R-aY-cpFP1.
